# Niemann-Pick type C2 protein supplementation in experimental non-alcoholic fatty liver disease

**DOI:** 10.1371/journal.pone.0192728

**Published:** 2018-03-09

**Authors:** Claus Uhrenholt Christensen, Emilie Glavind, Karen Louise Thomsen, Yong Ook Kim, Sara Heebøll, Detlef Schuppan, Stephen Hamilton-Dutoit, Christian Würtz Heegaard, Henning Grønbæk

**Affiliations:** 1 Department of Hepatology and Gastroenterology, Aarhus University Hospital, Aarhus, Denmark; 2 Institute for Translational Immunology and Research Center for Immunotherapy (FZI), University Medical Center, Johannes Gutenberg University, Mainz, Germany; 3 Division of Gastroenterology, Beth Israel Deaconess Medical Center, Harvard Medical School, Boston, Massachusetts, United States of America; 4 Institute of Pathology, Aarhus University Hospital, Aarhus, Denmark; 5 Department of Molecular Biology and Genetics, Aarhus University, Aarhus, Denmark; Medizinische Fakultat der RWTH Aachen, GERMANY

## Abstract

**Background and aims:**

Hepatic cholesterol deposition drives inflammation and fibrosis in non-alcoholic steatohepatitis (NASH). The Niemann-Pick type C2 (NPC2) protein plays an important role in regulating intracellular cholesterol trafficking and homeostasis. We hypothesized that intravenous NPC2 supplementation reduces cholesterol accumulation, hepatic inflammation and fibrogenesis in a nutritional NASH rat model.

**Methods:**

Rats were fed a high-fat, high-cholesterol (HFHC) diet for four weeks resulting in moderately severe NASH. Animals were treated with intravenous NPC2 or placebo twice weekly for either the last two weeks or the entire four weeks. End-points were liver/body- and spleen/body weight ratios, histopathological NASH scores, fibrosis, serum liver enzymes, cholesterol, lipoproteins, cytokines, and quantitative polymerase chain reaction derived hepatic gene expression related to cholesterol metabolism, inflammation, and fibrosis.

**Results:**

HFHC rats developed hepatomegaly, non-fibrotic NASH histopathology, elevated liver enzymes, serum cholesterol, and pro-inflammatory cytokines. Their sterol regulatory element binding factor 2 (SREBF2) and low-density lipoprotein receptor (LDL-R) mRNAs were down-regulated compared with rats on standard chow. NPC2 did not improve liver weight, histopathology, levels of serum liver enzymes or pro-inflammatory tumor necrosis factor-α (TNFα), Interleukin (IL)-6, or IL-1β in HFHC rats. Two weeks of NPC2 treatment lowered hepatic TNFα and COL1A1 mRNA expression. However, this effect was ultimately reversed following additional two weeks of treatment. Four weeks NPC2 treatment of rats raised ATP-binding cassette A1 (*ABCA1)* and low-density lipoprotein receptor (*LDLR)* mRNAs in the liver, concurrent with a strong tendency towards higher serum high-density lipoprotein (HDL). Furthermore, the peroxisome proliferator activated receptor-ɣ (*PPARG)* gene expression was reduced.

**Conclusions:**

NPC2 proved inefficient at modifying robust hepatic NASH end-points in a HFHC NASH model. Nonetheless, our data suggest that hepatic *ABCA1* expression and reverse cholesterol transport were upregulated by NPC2 treatment, thus presenting putative therapeutic effects in diseases associated with deregulated lipid metabolism.

## Introduction

Nonalcoholic fatty liver disease (NAFLD) is a continuum from simple steatosis to non-alcoholic steatohepatitis (NASH) characterized by inflammation and fibrosis of the liver, and may progress to cirrhosis. The prevalence of NAFLD in the general population of Western countries is 20–30%, and NASH affects 3–5% of the general adult population and up to 20–40% of obese and diabetic patients[1,2].

There is currently no efficient medical treatment for NAFLD, with the exception of lifestyle interventions; thus, reagents able to reverse biochemical and histopathological changes of NAFLD are highly warranted. A growing body of experimental and clinical data including epidemiological studies suggest that increased cholesterol intake and subsequent aberrant hepatocyte cholesterol metabolism and cholesterol accumulation play a significant role in NAFLD and NASH development and progression[3].

Niemann-Pick type C (NPC) disease is an autosomal recessive lysosomal cholesterol storage disorder[4]. Mutations in the major disease locus Niemann Pick C1 (*NPC1*) are the most prevalent cause of NPC disease (95%), the remaining resulting from mutations in the minor disease locus Niemann Pick C2 (*NPC2*), encoding the NPC2 protein[5]. NPC2 protein is a soluble cholesterol binding glycoprotein ubiquitously expressed throughout the body. Intracellularly, NPC2 mainly resides within late endosomes and lysosomal (LE/LY) compartments. NPC2 binds cellular cholesterol derived from the lipoprotein endocytic pathway and facilitates cholesterol egress out of LE/LY compartments into the cytoplasm. This transport is mechanistically coordinated with the lysosomal transmembrane protein NPC1 in bringing cholesterol to metabolically active sites within the cell. Additionally, NPC2 participates independently of NPC1 in cellular cholesterol export by interacting with the adenosine tri-phosphate (ATP) binding cassette A1 (ABCA1), the rate-limiting enzyme in high-density lipoprotein (HDL) particle formation[6]. Further, NPC2 secreted into the bile canaliculi stimulates ATP binding cassette G5 and G8 (ABCG5/8)-mediated cholesterol secretion[7].

Endocytosis of extracellular NPC2 protein imparts functional properties to NPC2-deficient cells in the same manner as the endogenously expressed protein *in vitro*[8] and *in vivo*[9]. The latter is evident by the significant improvement in liver and spleen steatosis seen following intravenous NPC2 administration in a NPC2 hypomorphic disease mouse model[9].

The aim of the present study was to investigate the effects of NPC2 treatment using a high-fat, high-cholesterol (HFHC) diet rat NASH model. We hypothesized that intravenous NPC2 administration would mobilize compartmentalized excess cholesterol trapped in hepatic cells within LE/LY compartments through SREBP2-pathway controlled alterations in gene expression, thereby ameliorating NASH changes. To test our hypothesis, we analyzed the liver weight/body weight ratio, spleen weight/body weight ratio, liver histopathology and fibrosis, serum liver enzymes, cholesterol lipoproteins, cytokines, and gene expressions related to cholesterol metabolism, inflammation, and fibrosis.

To assess the translatability of the data derived from the rat NASH model, we additionally compared gene expressions related to inflammation, fibrosis and cholesterol metabolism in a human population subjects with NAFLD (N = 14) or NASH (N = 12) with (N = 7) or without (N = 19) liver fibrosis. Other characteristics for these patients are as previously described[10].

## Methods

### Animals and design

Thirty-six female Wistar rats with a body weight of 180 g were sourced from Taconic (Ry, Denmark) and housed in Specific Pathogen Free environment at 21°C ± 2°C with three animals per cage at the Department of Animal Care at Aarhus University. All rats were of good health on arrival. The rats housed at the department undergo half-yearly serology to detect the presence of transmissible infections, and the facility remains free of transmissible disease. After one week of acclimatization on a standard isocaloric diet, we randomized the animals into four groups. Experimental NASH was induced by feeding the animals a HFHC diet (Research Diets Inc., New Brunswick, NJ, USA, D09052204) ad libitum for four weeks containing 39 gross margin percentage (gm%) fat (main components 9.5 gm% palmitic acid; 13.1 gm% stearic acid, 12.5 gm% oleic acid; and 3 gm% linoleic acid), 27 gm% protein, 19 gm% carbohydrates, and 2 gm% cholesterol. Controls were fed a standard isocaloric diet (Research Diets Inc.; D12450J). Animals had free access to tap water. Animals were observed on a day-to-day basis and post-injections for signs of infection or systemic inflammation (loss of hair, piloerection, huddled posture and decreased daily activity). This study was carried out in accordance with the recommendations in the Guide for the Care and Use of Laboratory Animals of the National Institutes of Health. The Danish Animal Experiments Inspectorate approved the experimental protocol (Permit number: 2013-15-2934-00971).

The study included four groups of nine animals each:

1. Isocaloric controls: Rats fed a standard diet and injected twice weekly with phosphate buffered saline (PBS) as placebo for four weeks;
2. HFHC controls: Rats fed HFHC diet and injected twice weekly with PBS as placebo for four weeks;
3. Two-week NPC2 (treatment group): Rats fed HFHC diet and injected twice weekly with PBS as placebo for two weeks and then twice weekly with NPC2 for two weeks;
4. Four-week NPC2 (prevention group): Rats fed HFHC diet and injected twice weekly with NPC2 for four weeks.

NPC2 was purified from bovine milk as previously described[9]. Aliquots with a concentration of 1.8 mg/mL were stored at -20°C. We injected 5 mg/kg NPC2 or the equivalent volume of vehicle (PBS) intravenously through the lateral tail veins, following Isoflurane (Forane^®^) anesthesia.

At the end of the study, and after overnight fast, we anaesthetized the animals with a subcutaneous injection of fentanyl/fluanisone (Hypnorm^®^, Jansen Pharma, Birkerød, Denmark) at 0.5 mL/kg and midazolam (Dormicum^®^, La Roche, Basel, Switzerland) at 2.5 mg/kg followed by retrobulbar whole blood extraction. The animals were sacrificed by cervical dislocation. Livers and spleens were weighed. Liver tissue samples from the left lobe were stored in 10% formalin for a maximum of 48h before paraffin embedding. Liver tissue was also snap-frozen in liquid nitrogen and stored at -80°C.

### Histology

Each liver specimen was evaluated by scanning at low-power, before detailed examination in five medium-power fields (20x objective). An experienced pathologist (SHD) graded NAFLD changes (NAFLD activity score (NAS); 0–11) using a modified version of the system described by Kleiner et al.[11]. The following parameters were assessed semi-quantitatively: steatosis (0–3), lobular inflammation (0–2), hepatocyte ballooning (0–2), and fibrosis (0–4). We assessed fibrosis on both hematoxylin/eosin and Masson trichrome stainings. We classified steatosis into three subtypes: macrovesicular, large droplet; macrovesicular, small droplet; and microvesicular.

### Blood analyses

Serum total cholesterol and high density lipoprotein (HDL)-cholesterol, triglycerides, alanine aminotransferase (ALT), aspartate aminotransferase (AST), gamma glutamyl transferase (GGT) and bilirubin were determined using enzymatic colorimetric assays based on the Cobas c-system. The sum of serum very low-density lipoprotein and low-density lipoprotein (VLDL+LDL) levels was assessed using the Friedewald formula[12], as the LDL concentration alone cannot be reliably determined in sera from hypercholesterolemic rats[13].

We determined serum tumor necrosis factor-α (TNF-α), interleukin (IL) -6, IL-1β, IL-10, and interferon-γ (IFN-γ) using a MULTI-SPOT Assay System “Proinflammatory Panel 1 (rat) kit” (MSD, Ballerup, Denmark).

### Liver tissue analyses

#### Ribonucleic acids (RNA) isolation and reverse transcription

Liver tissue was homogenized on a Tissuelyzer II (Qiagen, Hilden, Germany) and suspended in Ribozol^™^ (Amresco Inc., OH, USA) phenol reagent. Chloroform was added and the samples centrifuged, thus separating the proteinaceous organic phase and the aqueous nucleic acids containing interphase. The aqueous phase was mixed with an equivalent volume isopropanol. Finally, samples were washed three times in 70% ethanol/diethylpyrocarbonate (DEPC)-water, dried over laminar flow and the RNA resuspended in DEPC-water.

The final RNA concentrations were determined using an Infinite^®^ 200 Nanoquant (Tecan, Männedorf, Switzerland) and normalized to 1000 ng/μL. We synthesized complementary deoxyribonucleic acids (cDNA) with SuperScript^®^ Reverse Transcriptase (Thermo Fischer) on a MyCycler thermal cycler (Bio-Rad Laboratories, Hercules, CA, USA) according to the manufacturer’s protocol.

### Reverse transcriptase quantitative polymerase chain reaction (RT-qPCR)

RT-qPCR was performed on a 96-well StepOnePlus^™^ Real-Time PCR System (Life Technologies, Darmstadt, Germany) using TaqMan Gene Expression Assays (S1 Table). Samples were duplicated and the mean cycle threshold (C_T_) value used for statistical analysis. Gene expression was standardized using glyceraldehyde 3-phosphate dehydrogenase as house-keeping gene and data analyzed using the delta-delta-Ct method as described by Livak et al.[14]. For each gene, the C_T_ expression of isocaloric control animal number 1 was set as reference and the relative expressional levels compared with this sample.

### Microarray analysis of gene expression in human fatty livers

GeneChip data conducted in relation to a previously published clinical study was performed as previously described[10]. In brief, patients were differentiated between simple steatosis (NAFLD without NASH) or steatohepatitis (NASH) according to the FLIP algorithm[15] and the fibrosis score graded according to Kleiner et al.[11].

### Statistical methods

Data were analyzed using Kruskal-Wallis one-way analysis of variance by ranks followed by the Wilcoxon Rank Sum Test (Mann-Whitney) and presented as median with interquartile range. The NAS and components thereof, being discrete numerical, were compared parametrically with Welch approximation and presented as mean ± standard deviation. Differences were considered statistically significant for P< 0.05. We conducted all statistical analyses using STATA version 12 for Windows (Statacorp, TX, USA).

## Results

### Animal weights

The HFHC diet led to markedly increased liver weights (13.0 vs. 6.5 g, P<0.001), liver/body weight ratios (5.9 vs. 2.8%, P<0.001), spleen weights (0.9 vs. 0.6 g, P<0.001), and spleen/body weight ratios (0.40 vs. 0.25%, P<0.005) compared with the standard diet (Table 1). In HFHC animals, neither of the NPC2 treatment regimens affected body-, liver- or spleen weights, or liver/body weight or spleen/body weight ratios.

**Table 1:** Animal characteristics and blood analyses at baseline and in placebo treated HFHC control group after four weeks. Two-week NPC2 treatment group was fed the HFHC diet and injected intravenously with PBS for two weeks followed by two weekly NPC2 injections. The four-week NPC2 prevention group was fed the HFHC diet for four weeks with twice weekly NPC2 injections.

	Isocaloric diet	High-Fat High-Cholesterol Diet		
	Controls	Controls	2-weeks NPC2	4-weeks NPC2
Body weight, baseline (g)	198.5 (194.2–202.0)	194.7 (192.4–196.5)	194.9 (191.8–196.6)	193.7 (193.2–203.4)
Body weight, sacrifice (g)	222.4 (217.7–232.9)	220.8 (212.4–229.2)	211.3 (209.9–215.7)	219.4 (215.9–220.8)
Weight gain (g)	20.8 (18.3–34.9)	25.8 (19.8–33.7)	19.2 (17.3–22.6)	22.6 (17.9–27.4)
Liver weight (g)	6.5 (6.2–6.5)	13.0 (12.4–13.3)*	12.3 (11.7–13.5)*	13.7 (13.6–14.3)*
Liver/Body weight (%)	2.8 (2.8–3.0)	5.9 (5.9–6.0)*	5.8 (5.6–6.0)*	6.3 (6.1–6.8)*
Spleen weight (g)	0.58 (0.54–0.60)	0.88 (0.79–0.96)*	0.81 (0.80–0.85)*	0.98 (0.88–1.02)*
Spleen/Body weight (%)	0.25 (0.23–0.27)	0.40 (0.36–0.44)*	0.39 (0.36–0.41)*	0.45 (0.39–0.51)*
ALT (U/L)	32 (30–40)	881 (385–1275)*	1251 (958–1554)*	1553 (1239–1858)*
AST (U/L)	61 (59–66)	1153 (810–2502)*	2044 (842–2733)*	2312 (864–3550)*
GGT (U/L)	5 (5–5)	16 (5–23)*	29 (5–161)*	17 (8–80)*
Bilirubin (mg/dL)	5 (5–5)	5 (5–17)*	5 (5–8)*	9 (4–12)*
Total Cholesterol (mmol/L)	1.3 (1.1–1.4)	8.5 (7.8–9.0)*	9.1 (8.1–11.1)*	10.5 (8.1–12.8)*
HDL Cholesterol (mmol/L)	1.2 (1.0–1.3)	1.8 (1.5–2.0)*	1.9 (1.8–2.2)*	2.2 (1.8–2.4)*
(V)LDL Cholesterol (mmol/L)	†	6.7 (5.7–7.4)*	6.9 (6.1–9.3)*	8.3 (6.3–10.1)*
Triglyceride (mmol/L)	0.4 (0.4–0.5)	0.4 (0.3–0.5)	0.5 (0.4–0.5)	0.5 (0.4–0.5)

ALT and AST: Serum alanine and aspartate aminotransferases; GGT: Gamma glutamyl transferase; HDL-Cholesterol: High-density lipoprotein cholesterol; V-VLDL: Summated very-low density lipoprotein + low-density lipoprotein cholesterol. Data are presented as median (interquartile range).

*: P < 0.05 compared with isocaloric controls.

^†^: Below lower threshold.

### Liver morphology and histopathology

**Fig 1:**
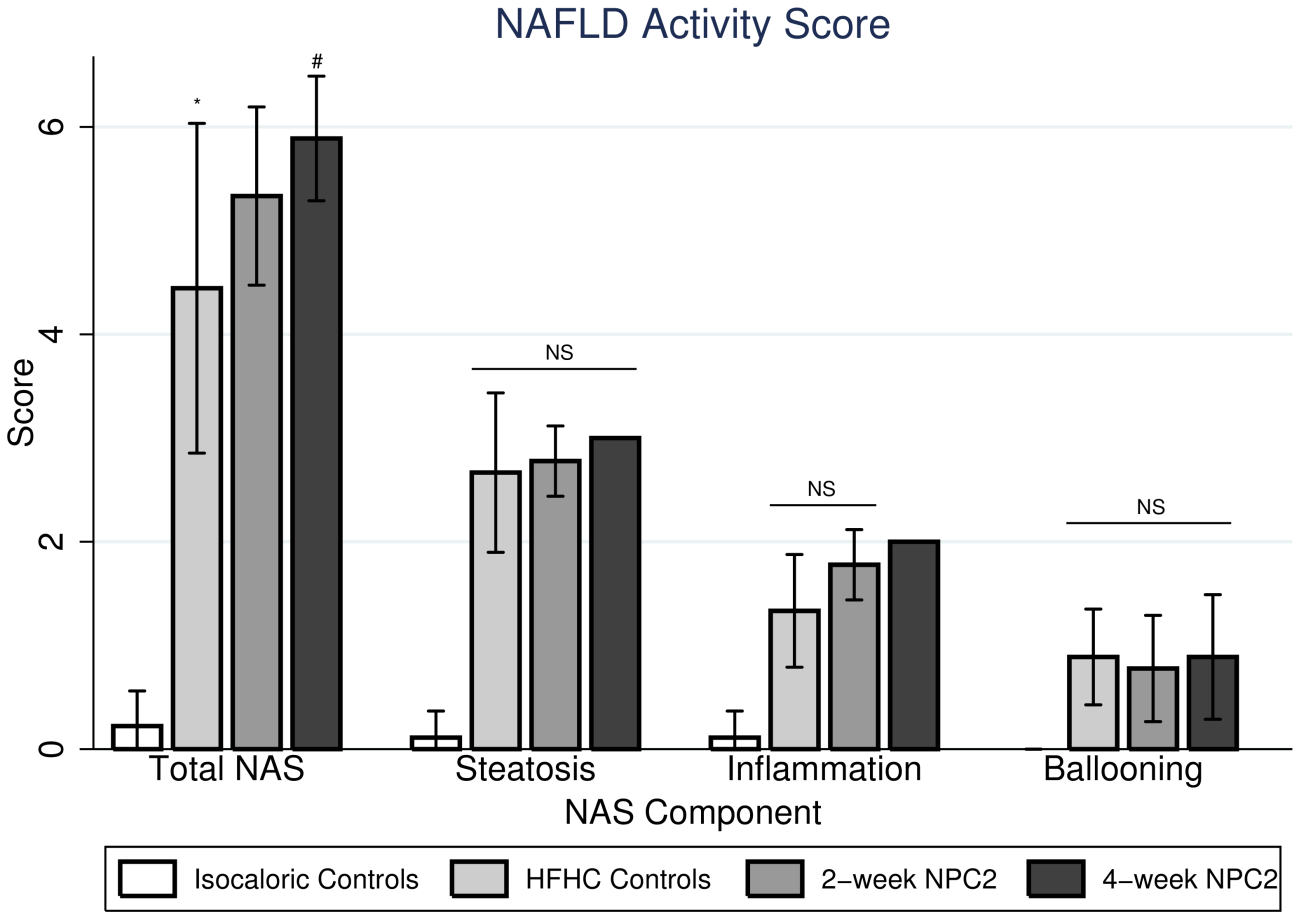
Hepatic histology scores. Hepatic histology scores graded semi-quantitatively according to Kleiner et. al. non-alcoholic fatty liver disease (NAFLD) activity score (NAS), steatosis (0–3), lobular inflammation (0–2), ballooning (0–2) and fibrosis (0–4) in isocaloric controls, high-fat, high-cholesterol (HFHC) controls, two-week NPC2 (treatment) and four-week NPC2 (prevention) animals. No animal had significant histological fibrosis on HE (Panel A) or Masson trichrome staining (Panel B). *: P < 0.05 compared with Isocaloric Controls. #: P < 0.05 compared with HFHC Controls.

**Fig 2:**
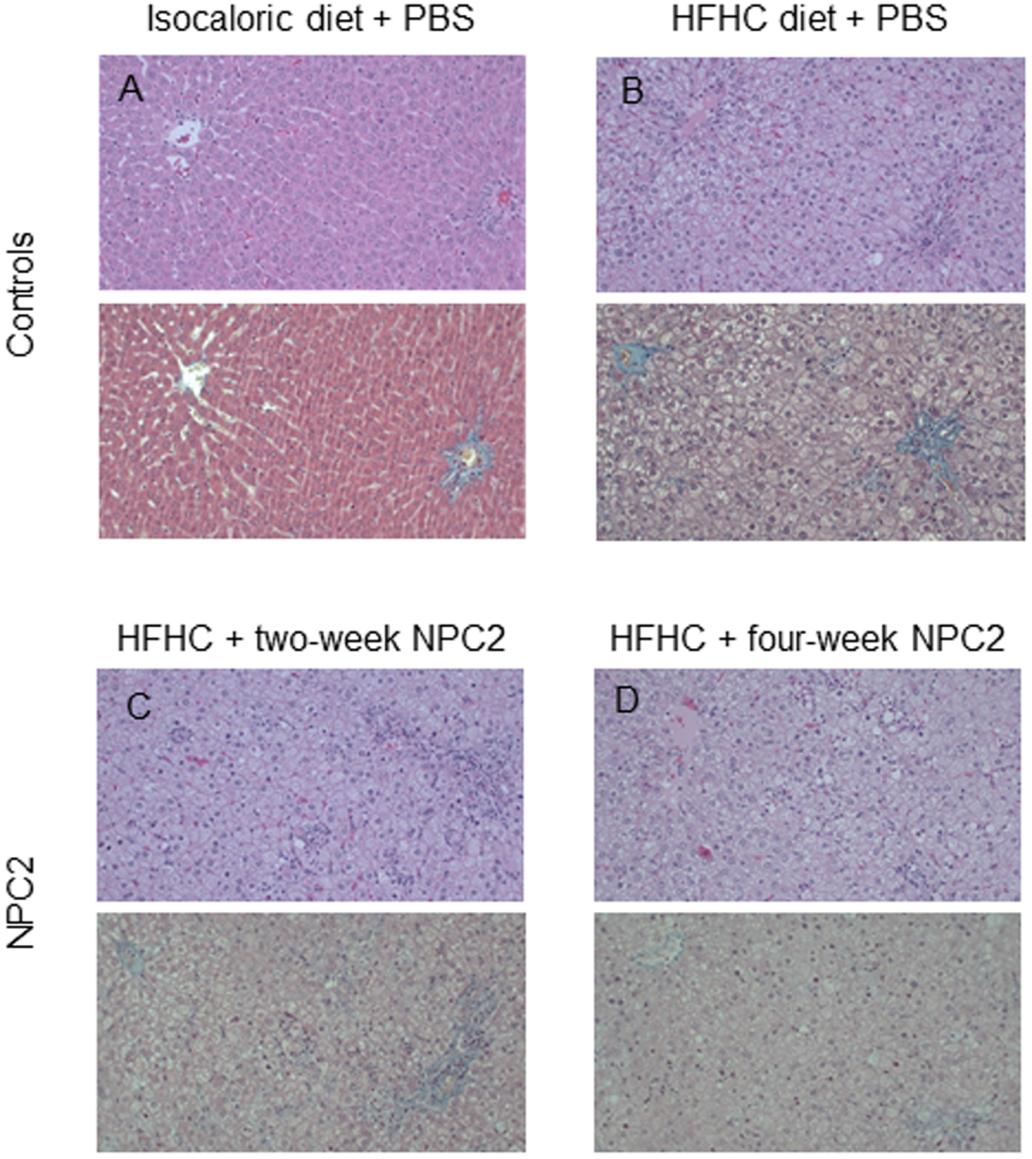
Representative histological pictures. (A) Rat fed control standard isocaloric diet with placebo PBS injections. Upper panel: Liver showing a normal histological picture. (Hematoxylin and eosin staining x 250). Lower panel: Liver with a portal tract (to the right) and a central area (to the left). There is normal histology without portal or perisinusoidal fibrosis. (Masson’s trichrome staining x 250). (B) Rat on HFHC diet with placebo PBS injections. Upper panel: The liver shows marked mixed, small and large droplet macrovesicular steatosis. (Hematoxylin and eosin staining x 250). Lower panel: Liver with a portal tract (to the right) and a central area (to the left). There is no evidence of either portal or perisinusoidal fibrosis. (Masson’s trichrome staining x 250). (C) Rat on HFHC diet with placebo PBS injections for two weeks, followed by NPC-2 injections for two weeks. Upper panel: Liver with marked mixed, small and large droplet macrovesicular steatosis and grade 2 lobular inflammation. (Hematoxylin and eosin staining x 250). Lower panel: The section shows a portal tract (to the right) and a central area (to the left). There is no evidence of either portal or perisinusoidal fibrosis. (Masson’s trichrome staining x 250). (D) Liver from a rat on HFHC diet with NPC2 injections for four weeks. Upper panel: There is marked mixed, small and large droplet macrovesicular steatosis with grade 2 lobular inflammation. (Hematoxylin and eosin staining x 250). Lower panel: The section shows a small portal tract (bottom right) and a central area (upper left). There is no evidence of either portal or perisinusoidal fibrosis. (Masson’s trichrome staining x 250).

Macroscopically, standard diet fed rats showed normal sized livers while HFHC rats presented with enlarged fat-infiltrated livers irrespective of treatment regimen (PBS, two- or four-week NPC2). Microscopically, the HFHC diet induced severe small droplet macrovesicular steatosis (mean score 2.7 ± 1.0), inflammation (mean score 1.3 ± 0.7), ballooning (mean score 0.9 ± 0.6), and increased the total NAS score (4.4 ± 2.1) compared with controls fed isocaloric diet (all P<0.0005) (Fig 1), but no fibrosis on either HE or MT stainings (data not shown). Neither NPC2 treatment regimen affected the degree of steatosis or ballooning when comparing with HFHC fed controls. While no effect of two-week NPC2 on inflammation or total NAS score was found, four-week NPC2 was significantly associated with inflammation (increased inflammatory foci; mean score 2.0 ± 0.0; P = 0.01), and tended to increase the total NAS score (5.9 ± 0.8; P = 0.07). Representative histological pictures are displayed in Fig 2.

### Blood analyses

The HFHC diet was associated with increased serum total cholesterol, VLDL+LDL (both P<0.001), HDL (P<0.005), ALT, and AST (P<0.0005) compared with the standard diet (Table 1). While NPC2 treatment of either duration did not affect total cholesterol or VLDL+LDL particle concentrations, four-week NPC2 treatment tended to increase serum HDL (2.2 vs. 1.8 mmol/L, P = 0.07) and triglycerides (0.48 vs. 0.39 mmol/L, P = 0.07). Two-week NPC2 treatment had no effect on ALT, but four-week NPC2 treatment tended to increase ALT levels (1551 vs. 881 mmol/L, P = 0.06).

### Cytokines

The HFHC diet significantly increased serum TNF-α, IL-6, IL-1β, and IL-10 levels. IFN-γ levels were unaffected compared with controls (Table 2). Neither two- nor four-week NPC2 treatment affected serum TNF-α, IL-6, IL-1β, or IL-10 levels.

**Table 2:** Serum pro- and anti-inflammatory cytokines (tumor necrosis factor-α (TNF-α), Interleukin (IL)-6, IL-1β, IL-10 and interferon-ɣ (IFN-ɣ)) at end of the treatment periods of 2 weeks after 2 weeks HFHC diet or 4 weeks on the HFHC diet.

	Isocaloric diet	HFHC Diet		
	Controls	Controls	2-weeks NPC2	4-weeks NPC2
TNF-α (pg/mL)	6 (5–7)	233 (152–256)*	243 (208–299)*	134 (117–166)*
IL-6 (pg/mL)	47 (41–53)	100 (83–178)*	140 (135–174)*	155 (103–176)*
IL-1β (pg/mL)	5 (5–5)	65 (52–113)*	69 (52–150)*	102 (85–151)*
IL-10 (pg/mL)	12.9 (10.7–13.4)	15.5 (14.5–18.4)*	16.0 (14.9–18.1)*	17.5 (14.2–18.1)*
IFN-ɣ pg/mL)	†	0.27 (0.05–0.96)	†	0.24 (0.05–1.13)

Data are presented as median (Interquartile range).

*: P < 0.05 compared with isocaloric controls.

^†^: Below lower threshold.

#### Hepatic messenger RNA (mRNA) expression related to inflammation and fibrosis

**Fig 3:**
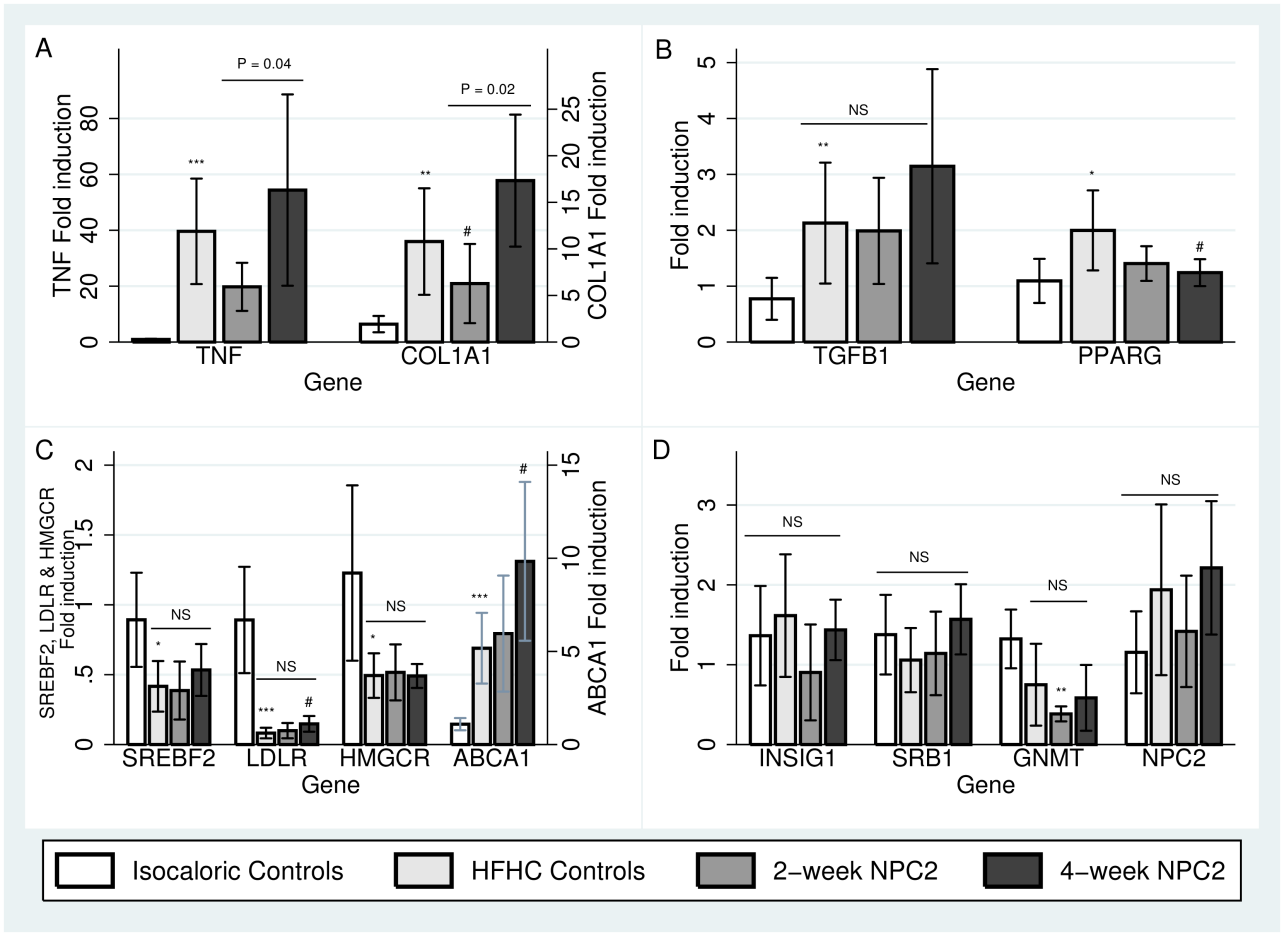
Hepatic gene expression related to inflammation, fibrogenesis and cholesterol metabolism. Relative mRNA expressions compared with isocaloric diet fed controls of *TNF* and *COL1A1* (Panel A), *TGFB1* and *PPARG* (Panel B), *SREBF2*, *LDLR*, *HMGCR* and *ABCA1* (Panel C) and *INSIG1*, *SRB1*, *GNMT* and *NPC2* (Panel D) in isocaloric controls, high-fat-high-cholesterol (HFHC) controls, two-week NPC2 (treatment) and four-week NPC2 (prevention) animals. Bars represent median ± interquartile range. *: P < 0.05 compared with Isocaloric Controls. **: P < 0.005 compared with Isocaloric controls. ***: P < 0.0005 compared with Isocaloric controls. #: P< 0.05 compared with HFHC Controls. ¤: P<0.05 compared with two-week NPC2.

The HFHC diet robustly up-regulated liver *TNF*, type 1 collagen A1 *(COL1A1)*, transforming growth factor beta-1 *(TGFB1)*, and peroxisome proliferator activated receptor gamma *(PPARG)* gene expression compared with isocaloric controls (Fig 3 Panels A and B).

Two-week NPC2 treatment tended to reduce hepatic *TNF* expression (P = 0.08), whereas four-week treatment had no effect compared with HFHC fed controls. Two-week NPC2 treatment significantly reduced *COL1A1* gene expression. This effect was not found with four-week NPC2 treatment. When comparing two- and four-week NPC2 treatment groups, the four-week NPC2 treated group showed higher gene expressions of both *TNF* and *COL1A1* compared with the two-week treated group (P = 0.04 and P = 0.02, respectively). NPC2 had no effect on the gene expressions of *TGFB1* or its pseudo-receptor bone morphogenetic protein and activin membrane-bound inhibitor (*BAMBI*, (data not shown). Four-week NPC2 treatment tended to reduce *PPARG* expression compared with the HFHC diet group (2.0 vs. 1.24 fold induction, P = 0.07).

#### Hepatic mRNA expression related to cholesterol metabolism

The HFHC diet suppressed expression of sterol regulatory element binding factor 2 (*SREBF2*) (P = 0.02), the low-density lipoprotein receptor *(LDLR)*, P<0.001), 3-hydroxy-3-methyl glutaryl CoA reductase *(HMGCR)* (P = 0.02); and tended to suppress glycine N-methyl transferase *(GNMT*, P = 0.07). In contrast, *ABCA1* gene expression was induced (P<0.001) compared with isocaloric controls (Fig 3, Panel C and D). *SREBF2*, *HMGCR*, and *GNMT* gene expressions were all unaffected by NPC2 treatment of either duration compared with HFHC fed controls, whereas four-week NPC2 treatment tended to induce *LDLR* expression (0.08 vs. 0.15 fold induction, P = 0.07). Similarly, four-week NPC2 treatment resulted in further induction of *ABCA1* compared with HFHC fed controls (9.8 vs. 5.2 fold induction, P = 0.03; Fig 3 Panel C). Insulin-induced gene 1 *(INSIG1)* and scavenger receptor type B1 *(SRB1)* gene expressions were similar in HFHC fed controls and isocaloric control rats. Both genes were unaffected by either NPC2 treatment regimen (Fig 3 Panel D). Endogenic *NPC2* gene expression tended to differ between all groups (P = 0.09).

#### Hepatic mRNA expressions in patients with NAFLD or NASH with or without fibrosis

When comparing patients with histological diagnoses of NAFLD and NASH, NASH patients had higher mRNA expressions *TGFB1* and *LDLR* expressions (respective up-regulations of 17 and 27%, both P < 0.05) and lower *GNMT* expression (down-regulated by 45%) (Table 3).

**Table 3:** Relative gene expressions in NAFLD and NASH patients and in fibrotic and non-fibrotic NAFLD (F1/2 versus F0).

	NAFLD compared with NASH			F0 compared with F1/2		
	Fold change	NASH relative to NAFLD	P	Fold change	F1/2 relative to F0	P
TNF	1,06	Up	0,24	1,00	Down	0,99
COL1A1	1,14	Up	0,08	1,12	Up	0,17
TGFB1	1,17*	Up	0,02	1,15	Up	0,06
PPARG	1,25	Up	0,10	1,20*	Up	0,02
BAMBI	1,03	Down	0,60	1,05	Down	0,50
SREBF2	1,15	Up	0,10	1,09	Up	0,34
LDLR	1,27*	Up	0,02	1,11	Up	0,38
HMGCR	1,30	Up	0,16	1,04	Up	0,83
GNMT	1,45*	Down	0,03	1,40	Down	0,08
ABCA1	1,06	Down	0,19	1,00	Down	0,92
INSIG1	1,25	Up	0,10	1,39*	Up	0,02
SRB1	1,03	Up	0,54	1,11*	Up	0,02
NPC1	1,09	Up	0,09	1,13*	Up	0,03
NPC2	1,12	Up	0,37	1,30*	Up	0,05

Relative mRNA expressions between patients with NAFLD and NASH (left) and between patients with or without liver fibrosis.

*: P < 0.05.

Livers of patients with fibrosis (all F1 apart from 1 patient whom was F2) had up-regulations of *PPARG* (20%), *INSIG1* (39%), *SRB1* (11%), *NPC1* (13%), and *NPC2* (30%) (all P < 0.05) (Table 3).

## Discussion

This is the first study to test NPC2 protein supplementation as a potential means to lower cholesterol accumulation and subsequent pathological inflammation and fibrosis in a HFHC experimental NASH model. In disagreement with our hypothesis, NPC2 treatment did not improve hepatomegaly or histopathology in HFHC diet-induced experimental NASH. In fact, four-week NPC2 treatment associated with slightly worse histopathology and tended to increase serum ALT indicating amplified hepatocyte injury. Further, four-week NPC2 suppressed hepatic *PPARG* and tended to induce *LDLR*. Two-week NPC2 led to favorable *COL1A1* suppression and similarly tended to suppress hepatic *TNF*, but these findings were inconsistent with the histopathology results and serum levels of TNF-α where no treatment effect was observed. Furthermore, *TNF* and *COL1A1* were significantly higher after four-week NPC2 treatment compared with two-weeks.

Previously, our group established that the mouse immune system does not induce significant humoral immune responses against bovine NPC2 protein[9]. This observation possibly will also apply for rats. Thus, we do not believe the lack of treatment effect to be due to anti-NPC2 antibodies.

The rat NASH model exhibited typical findings of NAFLD pathology including hepato- and splenomegaly, early NASH histopathology, hypercholesterolemia, increased serum liver enzymes, and increased pro-inflammatory cytokines as well as up-regulated hepatic *TNF*, *COL1A1*, and *TGFB1* gene expressions, similar to our previous studies using the same model[16–18]. Further, we observed changes in liver genes in the HFHC fed rats related to cholesterol homeostasis e.g. reduced mRNA expression levels of *SREBF2* alongside with *LDLR* and *HMGCR*, which are downstream molecules of SREBP2 [19,20]. However, there was no effect of NPC2 treatment on these cholesterol homeostasis genes.

The experimental NASH model comprised down-regulation of the SREBP2-pathway. This indicates that our NASH model comprises decreases in hepatic cholesterol biosynthesis and uptake most likely, by way of the high (2%) dietary cholesterol load. Further, the relatively short disease induction of four weeks may be too short a time span for the elevated IL-6, IL-1β to induce cholesterol biosynthesis as a previous study reported increased cholesterol biosynthesis after feeding a HFHC diet for 16 weeks[21].

These data contrasts data from human liver biopsies where NASH was associated with higher expressions of *SREBF2*, *LDLR* and *HMGCR* than simple steatosis, although, of the three, only *LDLR* was significantly increased in support of previous findings[22]. SREBP2 protein levels are elevated in human NASH, possibly due to a direct stimulatory effect of hyperinsulinemia[23] and high levels of circulating and hepatic IL-6 and IL-1β. These noxious stimuli induce cholesterol biosynthesis by up-regulating *SREBF2* and *HMGCR* gene expressions and increasing HMGCR enzymatic activity[24].

Reduced expression of *PPARG* and concomitant decrease in PPAR-γ protein levels and signaling in HSCs associates with progression of liver fibrosis and increases collagen production[20]. NPC2 treatment tended to down-regulate hepatic *PPARG* gene expression, concurring with *in vitro* results showing that NPC2 plays a role in PPAR-γ suppression[25]. We observed no effects of NPC2 treatment on genes related to fibrogenesis and no effects on liver fibrosis per se.

The notion of LDL receptor-mediated endocytosis as a protective mechanism in metabolic disorders such as NAFLD is underscored by the severely inflamed liver phenotype resembling NASH found in *LDLR* knock-out models[26]. The noxious element in these animals is probably the abundant uptake of lipoprotein particles into KCs mediated by members of the CD36 receptor superfamily. Scavenger receptors, which, unlike *LDLR*, are not subject to feedback regulation. We observed reduced LDLR expression in HFHC fed rats and observed that the four-week NPC2 treatment almost doubled the hepatic *LDLR* gene expression. However, for LDL receptor-mediated endocytosis to become hepato-protective this requires concomitant functional up-regulations in robust cellular cholesterol excretory pathways. This cannot have been the case, as we noticed no beneficial effect of NPC2 treatment on the serum levels of VLDL and LDL.

To the best of our knowledge, no studies have previously established how hepatic NPC2 is regulated in patients with NASH. While Liao et al. found no difference in serum levels of NPC2 between patients with fatty liver and healthy controls[27], they did report lower NPC2 protein levels in chronic viral hepatitis, cirrhosis, and hepatocellular carcinoma[28]. We found no difference in *NPC1* and *NPC2* gene expressions between NAFLD and NASH patients. However, both *NPC1* and *NPC2* were significantly up-regulated in biopsies from patients with liver fibrosis (F1-2) compared with patients without fibrosis. In light of the robust *GNMT* suppressions, we speculate that *NPC2* up-regulation might compensate for increased NPC2 protein decay due to GNMT deficiency in NASH.

In addition to its role in one-carbon metabolism, cytosolic GNMT doubles the half-life of NPC2[29]. The 2% cholesterol HFHC diet down-regulated hepatic *GNMT* gene expression. Likewise, *GNMT* was found significantly down-regulated in NASH patients livers compared to patients with NAFLD. And lastly, fibrotic NASH livers compared with non-fibrotic NASH livers tended to have decreased *GNMT* expressions. Thus, *GNMT* suppression could suggest that the model encompasses functional NPC2 deficiency secondary to GNMT suppression, similar to observations made in human fatty livers by other authors [29,30].

We demonstrated that NPC2 treatment raises total *ABCA1* mRNA levels. The importance of lysosomally-derived cholesterol in regulating ABCA1 expression has previously been demonstrated in the lysosomal cholesterol storage disorders Niemann Pick type C (NPC) and cholesteryl ester storage disease (CEST)[31,32]. In both cases the reduced flux of free cholesterol out of lysosomes lead to reduced 27-hydroxycholesterol production and reduced ABCA1 expression, the likely cause of low plasma HDL-cholesterol in both these disorders[33,34]. Delivery of exogenous oxysterols to NPC fibroblasts[35] and lysosomal cholesteryl ester to CESD fibroblasts[34] were both able to upregulate ABCA1 expression and cholesterol efflux to apoA1, suggesting that cytosolic cholesterol is a critical determinant of cholesterol-dependent ABCA1 gene regulation.

It has previously been shown that NPC2 replacement therapy enhances the rate of lysosome cholesterol efflux *in vitro* and *in vivo*[9] and that NPC2 is involved in directly transfer of lysosomal cholesterol to the mitochondrial outer and inner membranes[36]. The transfer of free cholesterol from the lysosome to the mitochondria potentially enhance 27-hydroxycholesterol synthesis, which upon binding to the nuclear receptor liver X receptor activates transcription of ABCA1[37]. We therefore believe that the enhanced delivery of cholesterol to mitochondria, brought on by the NPC2 interventions, brought on increases in *ABCA1* expression and serum HDL.

Recently, Twu et al.[38] demonstrated that NPC2 overexpression attenuates TGF-β sensitization by mobilizing cholesterol. The authors concluded that NPC2 might prove an effective agent against liver fibrosis progression. We found *TGFB1* up-regulated in NASH compared with NAFLD and in fatty livers with fibrosis compared with non-fibrotic NAFLD. We then investigated the hepatic expressions of *TGFB1* and *BAMBI* in the experimental rat NASH model and, contrary to our expectations, did not find any effect of NPC2 treatment on the expression of either of these genes.

Relatively little is known about how endogenous and exogenous NPC2 protein degrades. We know that the proteasome system can degrade NPC2 within the endoplasmic reticulum and that expression of the Nogo B receptor halts this process[39]. Further, Niemann Pick C1 Like 1 (NPC1L1) interacts with NPC2 in pre-lysosomal compartments and accelerates NPC2 protein break-down[40]. NPC1L1 however seems to have minimal effect on the half-life of endocytosed NPC2, which localizes within lysosomes[40]. Furthermore, glycine-N-methyl transferase interacts with and increases the half-life of NPC2 within the cytosol. Lastly, cathepsins may be partially responsible for exogenous NPC2 degradation[41]. Cathepsins are a group of cysteine proteases, which can degrade proteins taken up by endocytosis. Of particular interest, cathepsins B and L down-regulate NPC2 in pro-inflammatory macrophages *in vitro*[41]. Furthermore, cathepsin D is a potential substance which degrades NPC2. Cathepsin D is up-regulated as cholesterol accumulates in LE/LY compartments in an NPC1 knock-out model[42], and inhibition of cathepsin D in an *in vitro* NPC1 disease model ameliorates the cholesterol-storing phenotype[43]. Taking the NPC1-independent cholesterol-mobilizing effects of NPC2 into account[6], this could imply that also cathepsin D mediates degradation of exogenous NPC2.

In conclusion, the effect of NPC2 treatment on NASH remains equivocal. In our study using a HFHC NASH model, NPC2 treatment proved inefficient at modifying robust hepatic NASH end-points. However, NPC2 treatment seems to have accelerated post-lysosomal cholesterol transport through the induction of *ABCA1* and thereby increased HDL. The treatment-induced *PPARG* down-regulation and *LDLR* up-regulation in addition to adverse inductions of *TNF* and *COL1A1* after four weeks NPC2 treatment may explain the overall lack of improvement in NASH changes.

## Supporting information

S1 Table TaqMan Gene Expression Assays. TaqMan Gene Expression Assays and correlating lot numbers (Life Technologies, Darmstadt, Germany) used for quantitative real-time polymerase chain reaction.
(XLSX)

S2 Table Individual NAS histopathology scores. Individual NAS components (steatosis, inflammation, ballooning, fibrosis) for each animal graded by an experienced liver pathologist (SHD).
(XLSX)

S3 Table Blood analyses. Liver enzymes ALT, AST, GGT, bilirubin as well as cholesterols (HDL, LDL, total cholesterol determined by standard enzymatic colorimetric methods based on the cobas c-system.
(XLSX)

S4 Table Weights. Rat, liver and spleen weights, and liver/-body weight and spleen/body weight ratios.
(XLSX)

S5 Table Human NAFLD-NASH gene expression. Gene expression from the human NAFLD cohort.
(XLSX)

S1 Sheet Quantitative real-time polymerase chain reaction data. Cycle Threshold (CT) values and analysis conducted by the ΔΔCT method.
(XLSX)
